# A Ribonuclease Isolated from Wild *Ganoderma Lucidum* Suppressed Autophagy and Triggered Apoptosis in Colorectal Cancer Cells

**DOI:** 10.3389/fphar.2016.00217

**Published:** 2016-07-25

**Authors:** Xiuli Dan, Wenlong Liu, Jack H. Wong, Tzi B. Ng

**Affiliations:** ^1^School of Biomedical Sciences, Faculty of Medicine, The Chinese University of Hong KongHong Kong, China; ^2^Shenzhen Key Laboratory of Marine Biomedical Materials, Shenzhen Institutes of Advanced Technology, The Chinese Academy of SciencesShenzhen, China

**Keywords:** *Ganoderma lucidum*, ribonucleases, colorectal cancer cells, apoptosis, autophagy

## Abstract

The mushroom *Ganoderma lucidum* (*G. lucidum*) has been consumed in China as a medicine for promoting health and longevity for thousands of years. Due to its paramount and multiple pharmaceutical effects, *G. lucidum* has received considerable attention from researchers and its chemical constituents as well as their respective functions were gradually unveiled by using modern research methods. Herein, we reported the isolation of a protein (*Ganoderma lucidum* ribonuclease, GLR) with anti-colorectal cancer activities from *G. lucidum*. This protein is a 17.4-kDa RNA degrading enzyme (ribonuclease) and was purified by using liquid chromatography procedures. GLR manifested potent anti-proliferative and anti-colony formation activities on HT29 and HCT116 colorectal cancer cells by inducing cell cycle arrest in G1 phase through the regulation of cyclin D1 and P53 expression. GLR was demonstrated to induce cell apoptosis in HCT116 cells by activating unfolded protein response and caspase-9 regulated pathways. Besides, the ability to undergo autophagy which is a stress adaption mechanism to cope with metabolic crisis was significantly suppressed by GLR treatment in HCT116 cells. The activation of apoptosis in GLR-treated HT29 cells was, however, independent of caspase-9 and the suppression of autophagy was also relatively minor. Thus the apoptosis of HT29 cells triggered by GLR was much milder than that in HCT116 cells. Our findings show that the RNase from *G. lucidum* may be one of the bioactive components that contribute to the anti-colorectal cancer activity of *G. lucidum*.

## Introduction

*Ganoderma lucidum*, which is also called “Lingzhi” in Chinese, “Reishi” in Japanese, and “Youngzhi” in Korea, has been applied as traditional Chinese medicine to improve human vitality and longevity since 2000 years ago (Weng and Yen, 2010; Cheng and Sliva, 2015). Though its multiple functions have been recorded in the oldest Chinese medicinal book in 100 BC (Huie and Di, 2004), its pharmacological mechanisms have only been partially unveiled in recent decades. Laboratory and preclinical research has confirmed its anti-cancer, anti-bacterial, and anti-inflammatory activities as well as its modulating effects on immunity and cardiovascular diseases (Cheng and Sliva, 2015).

Triterpenoids and polysaccharides are the main constituents contributing to its anti-cancer functions (Weng and Yen, 2010; Ruan et al., 2015). Currently, there are more than 130 oxygenated triterpenoids and over 200 polysaccharides identified in *G. lucidum* (Huie and Di, 2004). Triterpenoids have a bitter flavor and share similarities with steroid hormones in structure. They exert anti-oxidative, anti-allergy and anti-hypertensive bioactivities (Huie and Di, 2004). Polysaccharides are macromolecules with diverse structures. Some of them are water-soluble while others are water-insoluble (Huie and Di, 2004). Many *G. lucidum* polysaccharides (GLPS) exert immune-modulating functions through activating the expression of cytokines associated with inflammatory response (like interleukin-1, interleukin-6, and tumor necrosis factor-α) or anti-tumor activity (like interferon-γ and tumor necrosis factor-α; Chen et al., 2004). Though there are reports indicating a direct cytotoxicity of GLPS on cancer cells (Liang et al., 2015), the anti-cancer functions of GLPS are, however, still generally believed to be closely associated with their immuno stimulating effects (Paterson, 2006).

In addition to triterpenoids and polysaccharides, the bioactive components of *G. lucidum* also include fatty acids, nucleosides, amino acids, proteins, peptides, alkaloids, steroids, and enzymes (Weng and Yen, 2010). The anti-cancer activity of ribonuclease (RNase), an RNA degrading enzyme, is the focus of the present paper. This type of protein is capable of catalyzing cleavage of phosphodiester bonds in RNA (Ardelt et al., 2009) and has been detected in different organisms, including animals, plants, bacteria, and fungi (Fang and Ng, 2011). RNases could be classified into two types, endoribonucleases and exoribonucleases, and each type could be further classified into different subtypes. RNases are not only important in clearing unwanted cellular RNA but also important in controlling gene expression, maturation, and turnover (Fang and Ng, 2011). After entrance to the cytoplasm, foreign RNase could degrade RNA and inhibit protein biosynthesis at both transcription and translation stages (Ardelt et al., 2009). Though mammalian cells usually contain a variety of ribonuclease inhibitors (RIs) to protect RNA from unwanted degradation, these human RIs, however, fail to inactivate the enzymatic activity of RNases from bacteria and fungi (Allgood et al., 1990; Makarov and Ilinskaya, 2003). The anti-tumor activity of RNases is directly associated with catalytic cleavage activity on ribosomes since catalytically inactive RNases are devoid of cytotoxicity. Among the widely studied RNases, onconase is the smallest member of the RNase A superfamily and demonstrates significant anti-tumor functions by inducing cell apoptosis independent of P53 protein (Lee, 2008). It has now been successfully applied to translational research clinically in the US and Europe (Lee, 2008), indicating a tremendous potential of RNases to be applied in cancer therapy.

## Materials and Methods

### Materials and Agents

*Ganoderma lucidum* was collected on the campus of The Chinese University of Hong Kong and authenticated by Prof. Shiuying Hu, Honorary Professor of Chinese Medicine, The Chinese University of Hong Kong. DEAE-Sepharose, Mono Q 5/50 GL, Superdex 75 10/300 GL columns and Akta Purifier were bought from GE Healthcare, UK. Primary antibodies against caspase-9, CHOP, IRE1 α, and PARP and secondary antibody for anti-rabbit lgG as well as anti-mouse lgG were purchased from Cell Signaling (Danvers, MA, USA). Primary antibodies against Apaf-1, ATF6, P62, and GAPDH were purchased from Abcam (Cambridge, UK). Primary antibodies against cyclin D1, LC3 and P53 were, respectively, purchased from Millipore (Billerica, MA, USA), Novus (Littleton, CO, USA) and Calbiochem (La Jolla, CA, USA). Click-iT EdU Imaging Kits were purchased from Invitrogen. Image-iT^TM^ LIVE Green Poly Caspases Detection Kit was obtained from Life Technologies (Carlsbad, CA, USA). WesternBrightTM ECL was purchased from Advansta (Menlo Park, CA, USA).

### Protein Purification

Fresh *G. lucidum* fruiting bodies (309 g) were first ground into a slurry in double-distilled water containing 0.2 M NaCl and stored at 4°C overnight before centrifugation at 14000 rpm for 30 min at 4°C. The supernatant was subjected to dialysis against double-distilled water to remove salt before lyophilization. The lyophilized powder was dissolved in 20 mM NH_4_HCO_3_ (pH 9.6) buffer and subsequently loaded on a DEAE-Sepharose column (5 cm × 17 cm; 340 ml bed volume) pre-equilibrated with the same buffer. Unadsorbed fraction was eluted with 20 mM NH_4_HCO_3_ (pH 9.6) buffer while the adsorbed fractions were eluted with the same buffer containing 0.2 M, 0.5 M, and 1 M NaCl successively. The bound fraction (eluted by 0.2 M NaCl) from DEAE-Sepharose was then loaded on a Mono Q column (5 mm × 50 mm; 1ml bed volume) pre-equilibrated with 20 mM NH_4_HCO_3_ buffer (pH 9.6) on a FPLC system. The unbound and bound proteins were, respectively, eluted with 20 mM NH_4_HCO_3_ buffer (pH 9.6) and the same buffer containing three successive linear gradients of NaCl (0-0.2 M in 5 ml, 0.2-0.3 M in 15 ml, 0.3-1 M in 5 ml). The fraction containing the target protein was collected and prepared for chromatography on a Superdex 75 HR10/300 column (10 mm × 300 mm; 24 ml bed volume) pre-equilibrated with 0.1 M NaCl in 20 mM Tris-HCl buffer on the FPLC system. The isolated protein from each step was dialyzed against water, lyophilized, dissolved in the corresponding buffer before chromatography on the next column.

### Sodium Dodecyl Sulfate-Polyacrylamide Gel Electrophoresis (SDS-PAGE) and Mass Spectrometric Analysis

Sodium dodecyl sulfate-polyacrylamide gel electrophoresis was carried out by using a 12.5% resolving gel and a 5% stacking gel. At the end of electrophoresis, the gel was stained with Coomassie Brilliant Blue and subsequently distained with 10% acetic acid until the background was clear. The molecular weight of the protein was estimated with reference to the molecular weight marker and subsequently confirmed by mass spectrometric analysis (UltrafleXtreme, Bruker). The purified protein was also trypsinized to produce small peptides by using in-gel digestion methods (Shevchenko et al., 2006) and analyzed by using a HPLC system (Ultimate 3000, Dionex) and a mass spectrometer (UltrafleXtreme, Bruker). The peptides were compared with those of reported fungal proteins in NCBI and SWISS-PROT data base.

### Assay of Ribonuclease Activity

The ribonuclease activity of the purified protein was tested by using the methods previously reported (Wang and Ng, 2006). Briefly, the tested sample was incubated with 200 μg yeast tRNA (Sigma) in 150 μl 100 mM MES buffer (pH 6.0) for 15 min before the reaction was terminated by the addition of 350 μl ice-cold perchloric acid (3.5%). The mixture was placed on ice for 15 min and was subsequently centrifuged to obtain the supernatant. The OD260 of the supernatant was measured and compared with the negative control.

### Assessment of pH and Thermal Stability

Purified protein was dissolved in buffer with different pH values (over the pH range of 1-12) for 30 min at room temperature and neutralized to pH 7 before ribonuclease activity was determined. The activity of protein treated with buffer at pH 7 was taken as the standard. To assess the thermal stability, protein was incubated at different temperatures (20°C to 100°C) for 30 min and subsequently fully cooled down to room temperature before the assay for ribonuclease activity. The activity of the protein at 30°C was taken as the standard.

### Cell Proliferation Assay

Colorectal carcinoma HCT116 cells and colorectal adenocarcinoma HT29 cells were cultured in RPMI-1640 medium with 10% fetal bovine serum (FBS) and 1% penicillin/streptomycin (PS) at 37°C. Cells (5 × 10^3^ cells in 100 μl per well) were seeded and cultured for 24 h in a 96-well microtiter plate before treatment with different concentrations of the purified RNase for another 24 or 48 h. Cell viability was then evaluated by the 3-(4, 5-dimethylthiazol-2yl)-2, 5-diphenyltetrazolium bromide (MTT) assay as described previously (Dan et al., 2015).

Cells (3 × 10^4^) were seeded on the coverslips in 24-well culture plates overnight before they were cultured in medium containing 1 μM GLR and 10 μM EdU for 24 h. Cells were then fixed in 3.7% formaldehyde in PBS for 10 min at room temperature, followed by permeabilization in 0.5% Triton X-100 for 20 min. The EdU was then detected by following the instructions of the Click-iT EdU imaging Kits (Invitrogen) by using Alexa Fluor^®^ 555 on a confocal microscope (Olympus FV1000-ZCD).

### Cell Colony Formation

Cells were seeded on a 6-well plate at the density of 2 × 10^3^ cells per well and treated with different concentrations of RNase for 48 h after cells had fully attached on the plate. At the end of the treatment, cells were rinsed with PBS, supplied with fresh medium and cultured for another 6 days, with medium refreshed every three days. Cell colonies were observed by using the ChemDoc XRS system after they had been fixed in 4% formaldehyde and stained with crystal violet (0.4 g/L). Colony size and numbers were compared between the wells treated with and without the purified RNase by using Image-Pro Plus 6.0.

### Detection of Poly-Caspases

The detection of polycaspase activation was performed by following the instruction of the manufacturer of Image-iT LIVE Green Poly Caspases Detection Kit. Briefly, cells were first treated with 1 μM purified RNase for 24 h and subsequently cultured in medium containing the reagent working solution for 60 min in a cell culture incubator. Cells were carefully rinsed with PBS, followed by incubation with 1 μM Hoechst 33342 for another 5 min in darkness. After removal of Hoechst 33342, cells were supplied with fresh PBS and observed by using a Nikon Live Cell Imaging System Ti-E.

### Characterization Using Flow Cytometry

Cell apoptosis was monitored by the Annexin-V and PI double staining method. Briefly, after treatment with RNase for 24 or 48 h, cells were collected and stained in binding buffer (0.01 M HEPES, pH 7.4, 140 mM NaCl and 25 mM CaCl_2_) containing 0.5 mg/ml Annexin V (BDPharmingen, San Jose, CA, USA) and 5 μg/ml PI (Sigma-Aldrich, St. Louis, MO, USA) in the dark for 15 min before application on a flow cytometer.

For cell cycle analysis, the treated cells were successively fixed in 70% ethanol on ice for 2 h, rinsed with enough PBS and stained with 50 μg/ml PI for 15 min in darkness. The samples were filtered through a nylon membrane before analysis on the flow cytometer.

### Western Blotting

Whole cell extracts prepared by using ice-cold RIPA lysis buffer (150 mM NaCl, 1% NP40, 0.5% sodium deoxycholate, 0.1% SDS, and 1 mM phenylmethylsulfonyl fluoride in 50 mM Tris-HCl buffer, pH 8.0) were applied for Western blotting with procedures as described previously (Dan et al., 2015). Briefly, supernatants of the cell extracts were loaded on SDS-PAGE for electrophoresis and subsequently transferred to a polyvinylidene fluoride membrane, followed by blocking in 5% milk. The membrane was then incubated with the first antibody overnight at 4°C and the second antibody at room temperature for 2 h. Bands were finally visualized by using WesternBrightTM ECL (Advansta).

### Assessment Using Transmission Electron Microscope (TEM)

Cells were collected after treatment with 1 μM GLR for 24 h and rinsed with PBS before fixation in 2.5% glutaraldehyde for 1 h. TEM Samples were prepared by using the method that has been previously reported (Dan et al., 2016). The prepared sample sections were successively stained with uranyl acetate and lead citrate before final examination with a transmission electron microscope (Hitachi H-7700).

### Cell Transfection

The pEGFP-LC3 expression plasmid was a generous gift from Professor Xiaoqiang Yao (School of Biomedical Sciences, The Chinese University of Hong Kong, Hong Kong). Briefly, pEGFP-LC3 (human) was purchased from Addgene and amplified by using DH5a. The plasmid was then transfected into cells by using Lipofectamine 2000 (Invitrogen) by following the manufacturer’s protocols. Six-four hours after transfection, cells were trypsinized, seeded on coverslips and cultured in complete medium overnight before treatment with 2 μM GLR for another 48h. Cells were fixed in 3.7% formaldehyde and viewed with a confocal fluorescence microscope (Olympus FV1000-ZCD).

### Statistical Analysis

Results were taken from three independent experiments (*n* = 3) and data were expressed as means ± standard deviation (SD). Scatter diagram and histograms were drawn using GraphPad Prim 5 (Graphpad Software, La Jolla, CA, USA). Students’ *t*-test and one-way ANOVA followed by Bonferronni *post hoc* test were, respectively, applied to the comparison between two groups and multiple group comparison. A *p*-value < 0.05 was considered as statistically significant: ^∗^*P* < 0.05; ^∗∗^*P* < 0.01; ^∗∗∗^*P* < 0.001 versus respective control groups.

## Results

### Protein Purification and Characterization

**FIGURE 1:**
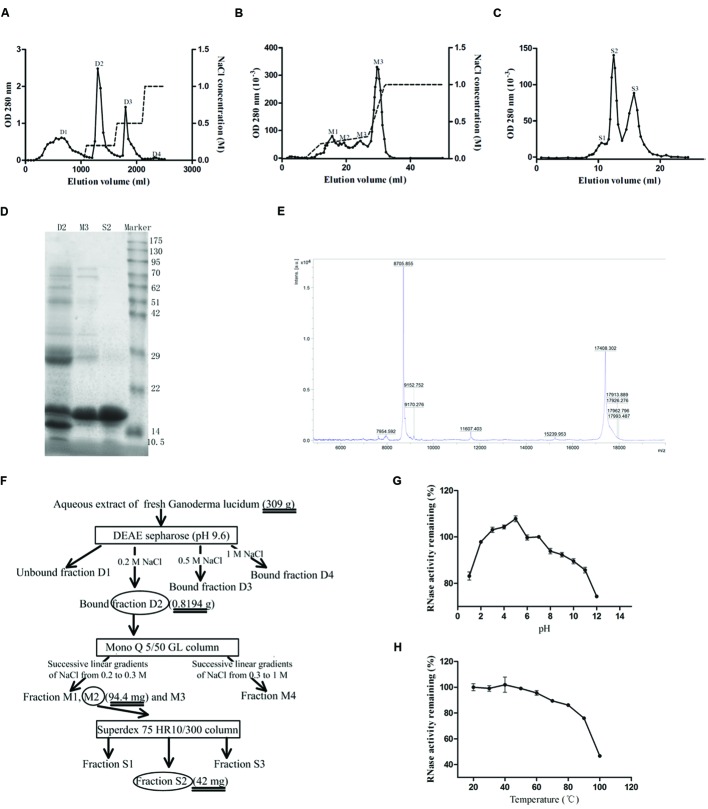
**Isolation of GLR. (A)** Aqueous extract of *Ganoderma lucidum* was first loaded on a DEAE-Sepharose column. The adsorbed fractions were obtained by eluting the column with different concentrations of NaCl (represented by the dashed lines). Peak D2 containing the highest ribonuclease activity was collected. **(B)** Adsorbed peak D2 was subsequently loaded on a Mono Q column and the adsorbed fractions were eluted with three successive linear gradients of NaCl (0-0.2 M in 5 ml, 0.2-0.3 M in 15 ml, and 0.3-1 M in 5 ml). **(C)** The only peak with ribonuclease activity, M3, collected in the previous step was subjected to Superdex 75 10/300 GL column to yield purified RNase (peak S2). **(D)** SDS-PAGE showing purity and molecular weight of GLR (peak S2). From the left to the right, the lanes represented the bound fraction D2 from DEAE-Sepharose, the bound fraction M3 from Mono Q, fraction S2 from Superdex 75 and molecular weight marker proteins, respectively. **(E)** Mass spectrometry results indicated that the acquired S2 fraction had a molecular weight of 17408.302 kDa. **(F)** Schematic representation of the chromatographic steps used for the purification. Square frame and ellipse, respectively, represented the applied column and collected fractions. **(G)** pH stability of GLR was measured after incubation in buffers of different pH values for 30 min at room temperature. **(H)** Thermal stability of GLR was tested after incubation at different temperatures. The remaining ribonuclease activity of treated GLR in **(G)** and **(H)** was tested by using yeast tRNA as substrate (*n* = 3).

The crude extract of *G. lucidum* was first separated into four fractions after chromatography on DEAE-Sepharose (**Figure 1A**). The second fraction (D2) eluted with 0.2 M NaCl contained the target protein after testing for ribonuclease activity. This fraction was then further fractionated by using a Mono Q column into four major fractions (M1, M2, M3, and M4) (**Figure 1B**). The third fraction M3 which exhibited ribounclease activity was collected for the next step of purification on a size exclusion Superdex 75 HR10/300 chromatography column. Based on the differences in molecular weight, proteins in fraction M3 were further resolved into three major fractions (S1, S2, and S3), as illustrated in **Figure 1C**. The second fraction S2 was demonstrated to have ribonuclease activity. Fraction S2 was then collected after dialysis against double-distilled water and lyophilization. The gel electrophoresis pattern (shown in **Figure 1D**) revealed that S2 contained only the target protein. According to the results of mass spectrometry which showed a peak at 17408.302 (**Figure 1E**), GLR had a molecular weight of 17.4 kDa. A yield of 42 mg purified RNase was obtained from 309 g fresh *G. lucidum*. A diagram illustrating the procedures is presented in **Figure 1F**. The purified RNase from *G. lucidum* was referred to as GLR in this text. The small peptides acquired from trypsinized GLR were dissimilar to any of those reported fungal proteins found in the database of Swiss-Prot and NCBI after mass spectrometry analysis.

GLR exhibited the highest ribonuclease activity in a weakly acidic environment (pH 5) and remained stable over a pH range of 2-9 (with more than 90% of activity remaining), as shown by **Figure 1G**. With reference to **Figure 1H**, the ribonuclease activity of GLR was intact up to 50°C and it was attenuated by only 5 and 25% after exposure to 60 and 100°C, respectively.

### GLR Exhibited Cell Toxicity in Colorectal Cancer Cells

**FIGURE 2:**
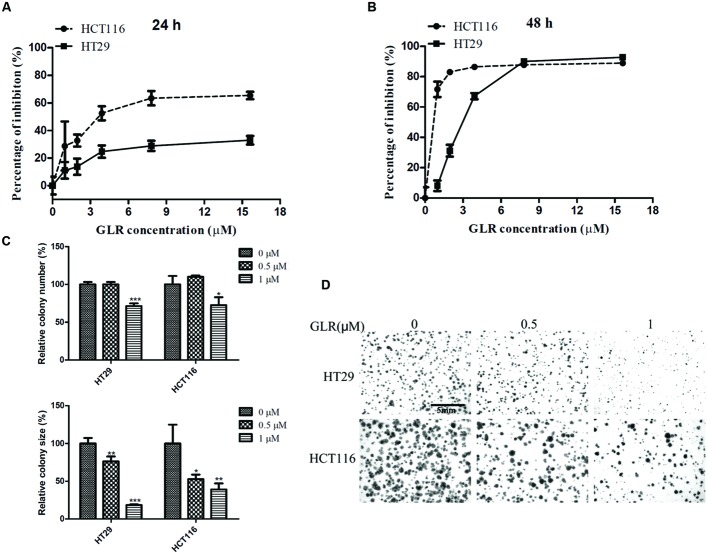
**Cytotoxicity of GLR on colorectal cancer cells.** HT29 cells and HCT116 cells were exposed to different concentrations of GLR for 24 h **(A)** and 48 h **(B)**. MTT assay was used to assess cell viability. In the colony formation assay, cells were first cultured with medium containing 0, 0.5, or 1 μM GLR for 48 h and subsequently allowed to grow in normal culture medium for 6 days. **(C)** The colony number (and colony size were monitored for comparison. **(D)** The representative images of colonies. ^∗^*P* < 0.05; ^∗∗^*P* < 0.01, ^∗∗∗^*P* < 0.001 versus respective control groups.

The viability of both HT29 and HCT116 cells was suppressed by treatment with GLR in a time- and dose-dependent manner. As illustrated in **Figure 2A**, GLR demonstrated a stronger inhibitory effect on the proliferation of HCT116 cells than that on HT29 cells at 24 h. At the time point of 48 h (**Figure 2B**), the divergence was still observable when cells were treated with GLR at a concentration lower than 9 μM. The IC_50_ (at 48 h) values for HT29 cells and HCT116 cells were, respectively, about 2.8 and 0.1 μM, indicating a higher sensitivity of HCT116 cells than HT29 cells to GLR treatment.

In keeping with results of cell proliferation assay, GLR treatment also strongly suppressed the colony forming ability of colorectal cancer cells. As shown in **Figures 2C,D**, the number of cell colonies was reduced by around 30% in both HT29 cells and HCT116 cell treated with 1 μM GLR. Besides, the colony sizes of both types of cells receiving the treatment of 1 μM GLR were reduced by more than 50% compared with that of the control.

### GLR Induced Cell Cycle Arrest at G1 Phase

**FIGURE 3:**
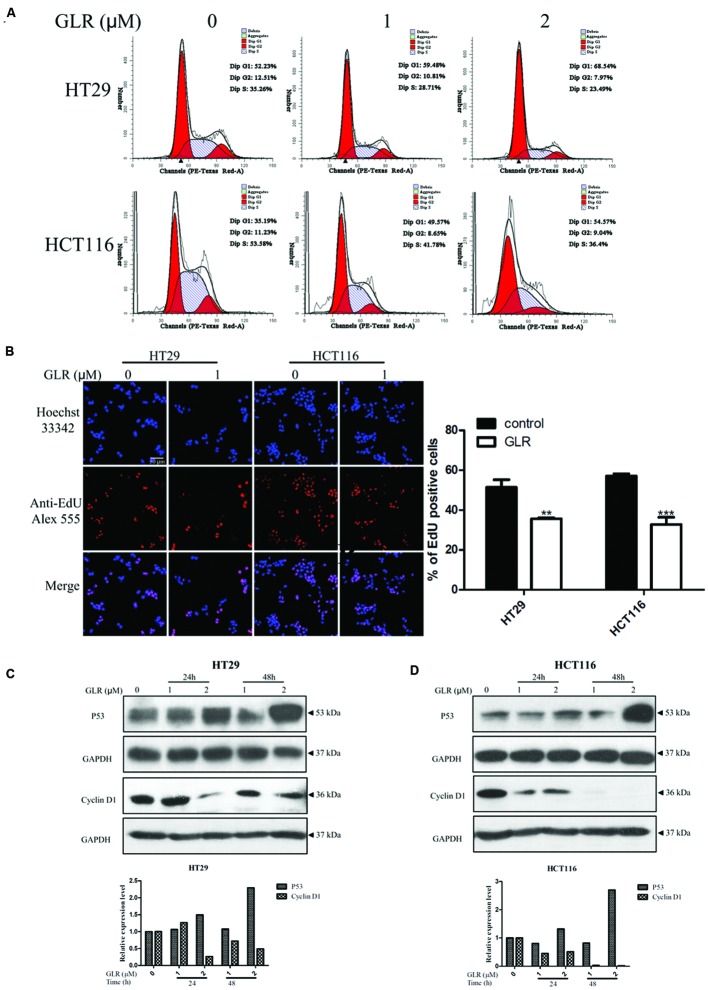
***Ganoderma lucidum* ribonuclease induced cell cycle arrest at G1 phase. (A)** HT29 and HCT116 cells were treated with different concentrations of GLR for 24 h. Cells were fixed in ice-cold ethanol and stained with PI for flow cytometric analysis. **(B)** Cells were seeded on the coverslips in a 24-well plate and cultured in the presence of 1 μM GLR and 10 μM EdU for 24 h. Cells were observed by using a confocal microscope after the labeling of Alexa Fluor^®^ 555. The percentage of EdU positive cells was displayed in the right panel (*n* = 3). ^∗∗^*P* < 0.01, ^∗∗∗^*P* < 0.001 versus respective control groups. **(C)** Western blotting analysis of cyclin D1 expression in HT29 **(C)** and HCT116 cells **(D)** was conducted after cells had been treated with indicated concentrations of GLR for 24 or 48 h. GAPDH was used as an internal control.

To elucidate the possible mechanism of the anti-proliferative activity of GLR on colorectal cancer cells, its modulatory effect on the cell cycle was monitored. The percentages of cells at different phases of the cell cycle were compared between cells cultured without GLR and cells cultured in the presence of different concentrations of GLR. As shown in **Figure 3A**, after colorectal cancer cells had been treated with 0, 1, or 2 μM GLR for 24 h, the percentage of cells in G1 phase increased from 52.23% (0 μM) to 59.48% (1 μM) or 68.54% (2 μM) in HT29 cells and from 35.19% (0 μM) to 49.57% (1 μM), or 54.57% (2 μM) in HCT116 cells, indicating a cell cycle arresting function of GLR. EdU labeling confirmed that GLR significantly reduced DNA synthesis of both types of colorectal cancer cells, as shown in **Figure 3B**.

In line with this result, the expression of cyclin D1 and P53 which play vital roles in cell proliferation was, respectively, downregulated and up-regulated in a time- and dose-dependent manner (**Figures 3C,D**).

### GLR Increased UPR

**FIGURE 4:**
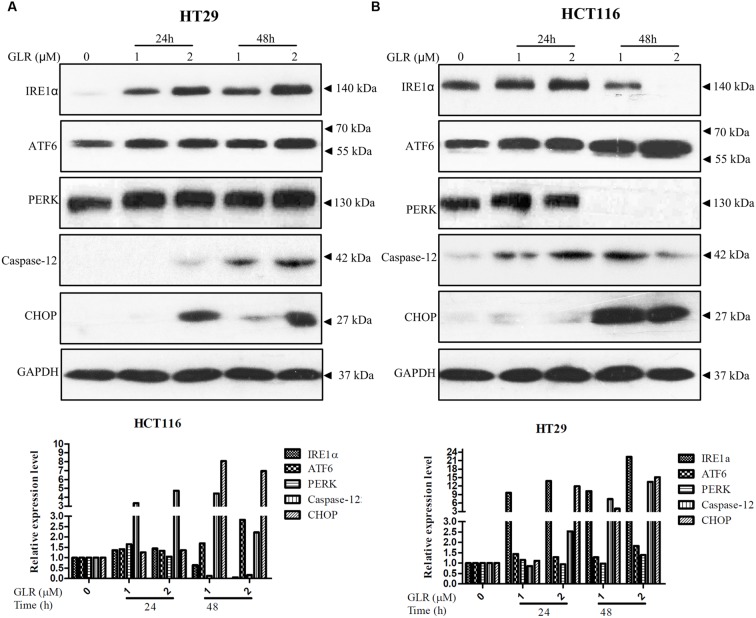
***Ganoderma lucidum* ribonuclease promoted UPR in colorectal cancer cells.** After treatment with GLR at the indicated concentrations for 24 or 48 h, the protein levels of IRE1α, ATF6, PERK, caspase-12, and CHOP in HT29 cells **(A)** and HCT116 cells **(B)** were determined by western blotting. GAPDH was used as an internal control.

The UPR is important for the homeostasis of protein-folding within the ER. IRE1α, activating transcription factor 6 (ATF6) and PKR-like ER kinase (PERK) are the three important sensor proteins for endoplasmic reticulum stress (ERS). They play vital roles in the activation of UPR by transmitting the information across the membrane to the cytosol (Gardner et al., 2013). These three stress sensors were upregulated in both HT29 and HCT116 cells treated with GLR, as displayed in **Figure 4**. However, the expression of IRE1α and PERK in GLR treated HCT116 cells was first up-regulated at the initial stage of treatment and then downregulated at 48h. Though UPR is a cytoprotective mechanism for cells to adapt to altered environments, UPR becomes cytotoxic and may induce cell apoptosis under conditions of severe and prolonged ER stress (Gardner et al., 2013). In the present scenario, the expression of CHOP which is one of the components of the ER stress-mediated apoptosis pathway (Oyadomari and Mori, 2004) was found to be upregulated (**Figure 4**), indicating a possibility of apoptosis-inducing effect of GLR on colorectal cancer cells.

### GLR Activated Apoptosis

**FIGURE 5:**
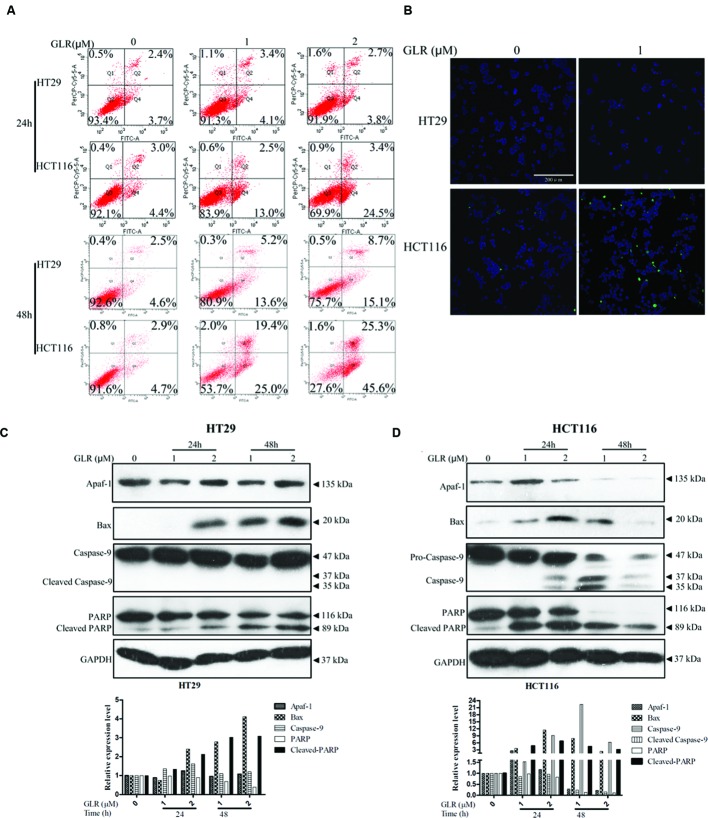
***Ganoderma lucidum* ribonuclease induced cell apoptosis in colorectal cancer cells. (A)** Cells exposed to the indicated concentrations of GLR for 24 or 48 h were harvested and subsequently stained with Annexin V/PI solution. Cells were then sorted by using flow cytometry. **(B)** Cells were treated with GLR at the indicated concentrations for 24 h, and nuclear morphological changes and caspase activation were, respectively, examined by staining with Hoechst 33342 and image-iT LIVE Green Poly Caspases staining reagent. Bars, 200 μm. Expression of Apaf-1, Bax, caspase-9, and PARP in HT29 cells **(C)** and HCT116 cells **(D)** was analyzed by western blotting after cells had been treated with different concentrations of GLR for 24 or 48 h. GAPDH was used as an internal control.

In order to confirm whether GLR treatment could induce cell apoptosis in colorectal cancer cells, Annexin V/PI staining was applied for evaluation of phosphatidylserine exposure. As shown in **Figure 5A**, for HT29 cells, no obvious difference in the percentage of apoptotic cells was observed between GLR-treated cells and untreated cells at the time point of 24h. However, in HCT116 cells, respectively 4.4, 13, and 25.4% of cells receiving 0, 1, and 2 μM GLR were undergoing early apoptosis at 24 h. At the time point of 48h in GLR-treated HT29 cells, around 19 and 24% of apoptotic cells treated with 1 and 2 μM GLR demonstrated apoptosis. For HCT116 cells exposed to 1 and 2 μM GLR, over 44 and 70% of the cells were found to undergo obvious apoptosis.

The activation of caspases under the stimulation of GLR was evaluated by using Image-iT LIVE Green Poly Caspases Detection Kit. After the cells had been treated with 1 μM GLR for 24 h, polycaspases signal was detected in GLR-treated HCT116 cells, but not in GLR-treated HT29 cells (**Figure 5B**). This result was consistent with those of the Annexin V/PI staining assay in that at the time point of 24 h, apoptosis was triggered only in GLR-treated HCT116 cells.

To reveal a more detailed activation process of cell apoptosis, we had monitored the expression of Apaf-1, apoptosis regulator BAX and an initiator caspase (caspase-9). Though BAX was upregulated by GLR treatment in both HT29 cells and HCT116 cells, the expression of Apaf-1 and activated caspase-9 was upregulated only in GLR-treated HCT116 cells (**Figures 5C,D**). Despite the inactivation of caspase-9, cleavage of PARP was still discernible in GLR-treated HT29 cells at 48 h, which indicated that apoptosis was also triggered in HT29 cells after treatment with GLR for a longer duration (48 h). Compared with HT29 cells, the cleavage of PARP in GLR-treated HCT116 cells was triggered at an earlier stage (24 h) and to a larger extent.

### GLR Suppressed Cell Autophagy

**FIGURE 6:**
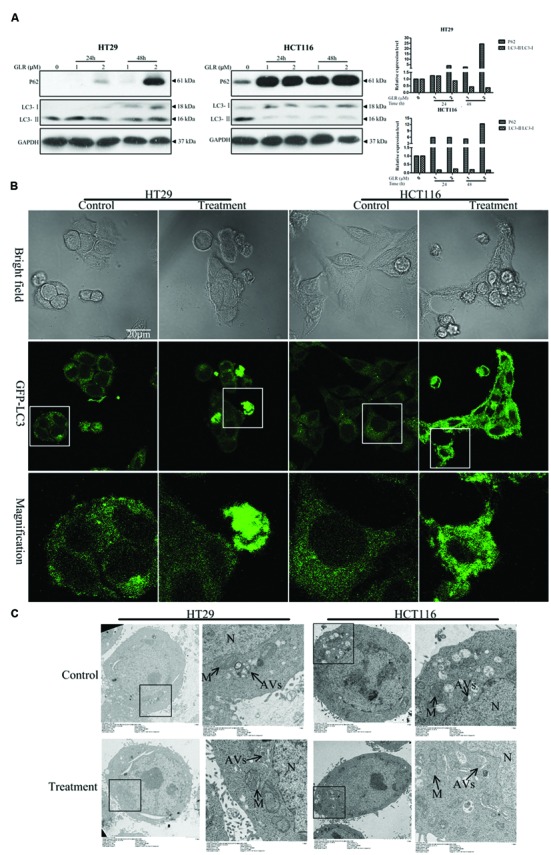
***Ganoderma lucidum* ribonuclease suppressed the activation of cell autophagy in colorectal cancer cells. (A)** HT29 cells and HCT116 cells were treated with the indicated concentrations of GLR for different durations before the protein expression levels of P62, LC3-I, and LC3-II were determined by western blotting. GAPDH was used as an internal control. **(B)** Subcellular expression and distributions of pEGFP-LC3. Colorectal cancer cells were transfected with the pEGFP-LC3 vectors in OPTI-MEM I Reduced Serum Medium for 6 h. After treatment with 2 μM GLR for 48 h, the fluorescence signals of cells were acquired by confocal microscopy. **(C)** Ultrastructures of HT29 cells and HCT116 cells treated with or without GLR. Cells were cultured with medium containing 0 or 1 μM GLR for 24 h before preparation for TEM sections. N, nucleus; M, mitochondrion; AVs, autophagic vacuoles.

Intriguingly, GLR was found to suppress cell autophagy in both HT29 cells and HCT116 cells. As evidenced in **Figure 6A**, accumulation of P62, upregulation of LC3-I and downregulation of LC3-II were detected in cells receiving GLR. These two phenomena were particularly conspicuous in HCT116 cells.

pEGFP-LC3 labeling results indicated greater abundance of LC3 protein in GLR-treated HCT116 cells and a portion of GLR-treated HT29 cells than in the respective control cells when the images were taken under the same conditions (**Figure 6B**). GLR-treated cells also showed more green fluorescent spots.

TEM analysis of autophagic vacuoles (AVs) (**Figure 6C**) in both types of colorectal cancer cells indicated that GLR treatment did not induce an increase of autophagic vacuoles in the cytoplasm of the cells.

## Discussion

In the present research, we purified a ribonuclease from *G. lucidum* through the application of chromatographic procedures. The molecular mass of GLR isolated in the present study was 17.4 kDa, which lies within the range 9.5 to 45 kDa reported for mushroom RNases (Kobayashi et al., 2003; Ngai et al., 2003). Though a least two RNases have been reported from *G. lucidum*, including a 42 kDa RNase (Wang et al., 2004) and an RNase with gene named RNGI30, GLR was dissimilar to any of the reported fungal proteins as judged by molecular weight or MS analysis. However, GLR resembled RNases from *Lyophyllum shimeiji* (Zhang et al., 2010), *Ramaria formosa* (Zhang et al., 2015), *Russulus virescens* (Wang and Ng, 2003), and *Thelephora ganbajun* (Wang and Ng, 2004) in adsorption on DEAE-cellulose and Q-ion exchangers, and differed from *Hypsizigus marmoreus* (Guan et al., 2007) and *Russula delica* RNases (Zhao et al., 2010) which were unadsorbed on DEAE-cellulose.

Ribonucleases have been found in a variety of organisms, encompassing plants (Green, 1994; Podzimek et al., 2011), fungi (Fang and Ng, 2011), bacteria (Aphahasenko et al., 1979), and animals (Newton et al., 1997). Some of the ribonucleases were reported to be immunosuppressive, embrytoxic, or aspermatogenic (Leland and Raines, 2001) and some of them demonstrated potent anti-tumor activity (Piccoli et al., 1999; Fang and Ng, 2011; Zhang et al., 2010; Fiorini et al., 2015). The present article constitutes the first report on induction of cell cycle arrest and apoptosis by *G. lucidum* RNase.

When HCT116 cells and HT29 cells were, respectively, treated with 3 and 8 μM GLR for 48h, proliferation of both types of cells was suppressed by more than 80%. At the early stage of treatment (24 h), the proliferation of HCT116 cells tended to be inhibited to a significantly higher degree than that of HT29 cells receiving the same dosage of GLR and this difference was attenuated but still discernible at 48 h. This result indicated a time and effect divergence of GLR’s inhibitory function on these two types of colorectal cancer cells. Further assessment indicated that GLR could induce cell cycle arrest in G1 phase by downregulating the expression of cyclin D1 and upregulating the expression of P53. The proto-oncogene protein cyclin D1 is overexpressed in a variety of cancers, including colon cancer (Arber et al., 1997), breast cancer (Lin et al., 2000), and lung cancer (Gautschi et al., 2007) etc. to advance cell cycle progression from G1 phase to S phase. The report of Arber et al. (1997) provided direct evidence for the critical role of cyclin D1 in maintaining the malignant phenotype of colon cancer cells and pointed out its potential application as a cancer marker and a target for cancer therapy. As evidenced in **Figures 3C,D**, cyclin D1 was indeed prominently detected in the untreated colorectal cancer cells and its expression was adjustable by GLR treatment, indicating the great potential of GLR to target the overexpressed cyclin D1 in colorectal cancer.

Though GLR triggered cell apoptosis in both HT29 cells and HCT116 cells, the apoptotic pathways in these two cells lines were, however, a little bit different. Cleavage of PARP in GLR-treated HT29 cells was conducted independently of caspase-9 and Apaf-1 (as shown in **Figure 5C**). PARP is a zinc-finger DNA-binding protein and it is implicated in the maintenance of genomic stability and DNA damage repair. Once it is cleaved by caspases which may be caspase-3 or caspase-7, it becomes inactive and loses the ability to respond to DNA damage and promotes cell apoptosis (Yang et al., 2004). The activation of caspase-3 which is upstream of PARP cleavage could be activated in a caspase-9 dependent and independent way (Porter and Janicke, 1999), which explains the possibility of PARP cleavage in the absence of caspase-9 activation. In the present case, the cleavage of PARP in GLR-treated HT29 cells could most probably be regulated through ER stress-mediated pathways which usually involve transcriptional activation of the gene for CHOP, activation of the cJUN NH2-terminal kinase (JNK) pathway or activation of caspase-12(Oyadomari and Mori, 2004). Among the aforesaid pathways, we have detected the upregulation of caspase-12 and CHOP in GLR-treated HT29 cells. Accordingly, the expression of three stress sensors IRE1α, ATF6 and PERK was also upregulated. Different from HT29 cells, HCT116 cells receiving the same treatment of GLR were found to undergo apoptosis by both activating caspase-9 and ER stress, which led to an earlier and more obvious cleavage of PARP than in GLR-treated HT29 cells.

Autophagy is a natural and destructive process that degrades unwanted or dysfunctional cellular proteins, organelles and bulk cytoplasm (Cuervo et al., 2005). It is a cellular response to a variety of stresses like nutritional starvation (Scott et al., 2004), hypoxia (Bellot et al., 2009), and drug treatment (Lei and Chang, 2007) etc. It could function both as a stress adaptive mechanism to avoid cell death and also promote programmed cell death type II (Lei and Chang, 2009). The specific role of autophagy in cell fate is context-dependent. Due to the continuing uncontrolled proliferation of cancer cells, the supply of sufficient energy and nutrition is critical, especially when the cancer cells are under the stimulation of metabolic stress, like starvation. In this case, autophagy is usually employed by cells to recycle cellular constituents from dysfunctional cells to survive metabolic stress and inhibition of autophagy could accelerate cell apoptosis (Boya et al., 2005). In the present study, suppression of autophagy was detected in both HT29 and HCT116 colorectal cancer cells after treatment with GLR, as observed in **Figure 6**. Accumulation of P62 was discerned as early as 24 h post-GLR treatment at the dosage of 1 μM in HCT116 cells while significant accumulation of P62 in HT29 cells was detected only at a later time point (48 h) and a higher concentration of GLR. Besides, downregulation of LC3-II in GLR-treated HCT116 cells was much more significant than that observed in GLR-treated HT29 cells. However, when LC3 was overexpressed in HT29 cells and HCT116 cells by using transfection vectors, the overall fluorescence signal of LC3 and fluorescent spots were more abundantly detected in GLR-treated cells than untreated cells. This phenomenon was again especially obvious in GLR-treated HCT116 while it was only observed in a portion of GLR-treated HT29 cells. The abundance of LC3 in GLR-treated cells may be explained by the blockage of autophagic degradation which led to accumulation of LC3 and P62. However, further studies are still needed to confirm this speculation. Generally, GLR induced more severe autophagy suppression in HCT116 cells than in HT29 cells and this may help explain why GLR triggered more potent apoptosis in HCT116 cells than it did in HT29 cells, which would then help explain why GLR exhibited a higher toxicity on HCT116 cells than HT29 cells. Then why did GLR trigger more severe autophagy dysfunction in HCT116 cells than HT29 cells? This may be attributed to the difference in protein transport efficiency or enzyme expression in the two cells lines.

To sum up, the RNase which was isolated from *G. lucidum* in the present investigation could undermine the proliferation of colorectal cancer cells by downregulating cyclin D1 and inducing cell cycle arrest. This Rnase was capable of triggering UPR and autophagy dysfunction, which then induced apoptosis in colorectal cancer cells.

## Author Contributions

TN conceived and designed the experiments. XD performed the experiments, analyzed the data, and wrote the manuscript. WL performed the experiments suggested by the reviewers, analyzed the data, and helped revise the manuscript. JW gave great advice in conducting the suggested experiments and helped check the writing.

## Conflict of Interest Statement

The authors declare that the research was conducted in the absence of any commercial or financial relationships that could be construed as a potential conflict of interest.

## Abbreviations

Apaf-1: apoptotic protease activating factor 1
CHOP: C/EBP homologous protein
ER: endoplasmic reticulum
FPLC: Fast protein liquid chromatography
GLR: *Ganoderma lucidum* ribonuclease
GLPS: *Ganoderma lucidum* polysaccharides
IRE1α: inositol requiring enzyme 1 α
PARP: poly (ADP-ribose) polymerase
PERK: pancreatic ER kinase (PKR)-like ER kinase
PI: propidium iodide
RNase: ribonuclease
UPR: unfolded protein response
